# Supplementary material for: Diffusion wavelets on connectome: Localizing the sources of diffusion mediating structure-function mapping using graph diffusion wavelets
Source: Netw Neurosci. 2025 Jun 27;9(2):777–97. doi: 10.1162/netn_a_00456 (PMC12226145; doi:10.1162/netn_a_00456)
Supplement: Supplementary file 1 [file netn-9-2-777-s001.pdf]

Diffusion Wavelets on Connectome: Supplemental Document

A. Scale selection

Donnat et al.[1] provides bounds for the scale, but does not directly define an optimal scale since optimality is determined by downstream task. We found that our learned diffusion scales are in accordance with their analytical results. We refer to their equation in 4th section for scale of diffusion wavelets: based on [1], which gives analytical bounds to the scales, we clamp the diffusion parameters in this range and observe that the model performance remains unaffected.

$$-\frac{\log(\gamma)}{\sqrt{\lambda_1 \lambda_N}} \leq s \leq \frac{\log(\eta)}{\sqrt{\lambda_1 \lambda_N}} \tag{S1}$$

where $\eta = 0.85$ and $\gamma = 0.95$ are recommended arbitrary constants resulting in bounds $(0.044, 4.417)$ and the bounds for scales (s) use the product of the values of the first non-zero and the last eigen values (λ_1 and λ_N). When the model is forced to move out of this range, the model performance decreases. This experiment validates the usage of backpropagation for node specific scale selection, since it is in accordance with mathematical result.

B. Diffusion Scale Initialization

In this section we try different initializations for diffusion scale instead of initializing all at 0. We use the uniform $(a = 0, b = 10)$ [2] and normal $(\mu = 5, \sigma = 2)$ initialization strategies. Even after different initializations we see that, after training, the distribution tends towards a power-law.All these experiments are conducted at 100 epochs.

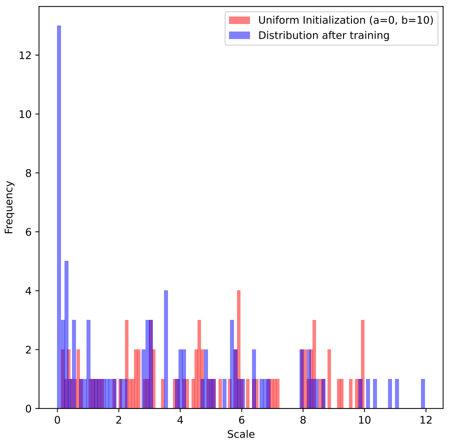

Fig. S1. The model is initialized with diffusion scales sampled from uniform distribution.

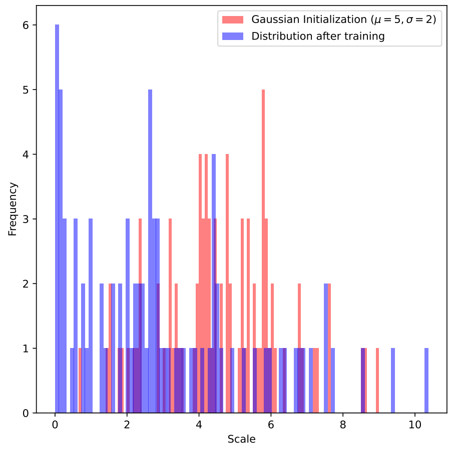

Fig. S2. The model is initialized with diffusion scales sampled from gaussian distribution.

C. Diffusion Scales for Top 10 Brain Regions

The Table S1 contains the brain regions having higher diffusion values. Most of the diffusion

Table S1. Top 10 Brain Regions by Diffusion Scale (Sorted High to Low)

Brain Region	Diffusion Scale
ctx-rh-frontalpole	0.207406
ctx-rh-bankssts	0.162382
ctx-lh-frontalpole	0.143831
ctx-lh-transversetemporal	0.099931
ctx-rh-pericalcarine	0.078568
ctx-rh-parsopercularis	0.077401
ctx-lh-pericalcarine	0.077179
ctx-rh-insula	0.071619
ctx-rh-transversetemporal	0.062333
ctx-lh-parahippocampal	0.058326

scales are small and lie close to each other. We list out the brain regions having higher diffusion values. These are diffusion scales learned and averaged over 5 independent runs.

D. Diffusion Scales for 7 resting state networks

The Figures S3 to S9 show the diffusion scales learnt for different resting state networks, Default Mode Network (DMN), Dorsal Attention Network (DAN), Limbic Network (LN), Somato Motor Network (SMN), Ventral Attention Network (VAN), Visual Network (VN) and Fronto Parietal Network (FPN) respectively.

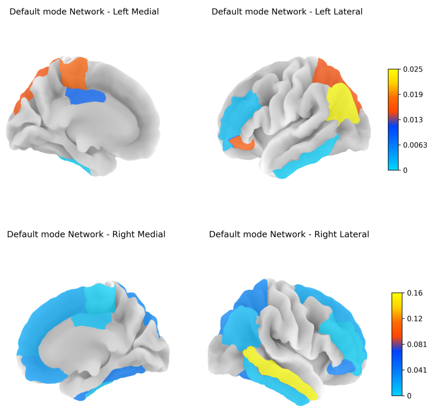

Fig. S3. Scales overlaid on parcellated surface mesh for Default Mode Network.

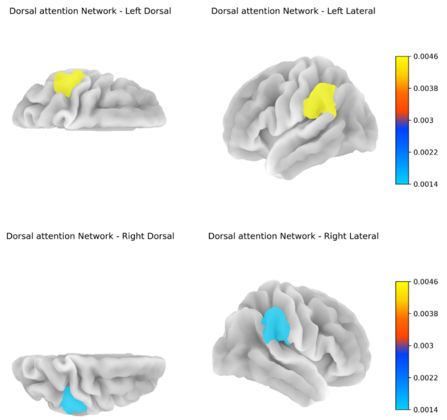

Fig. S4. Scales overlaid on parcellated surface mesh for Dorsal Attention Network.

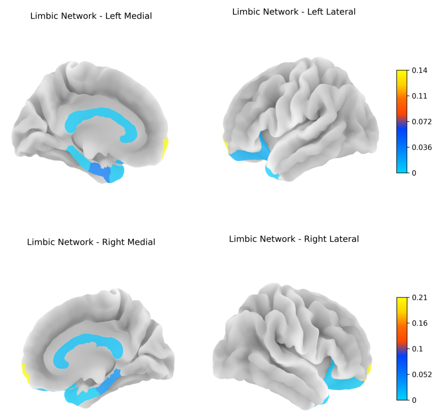

Fig. S5. Scales overlaid on parcellated surface mesh for Limbic Network

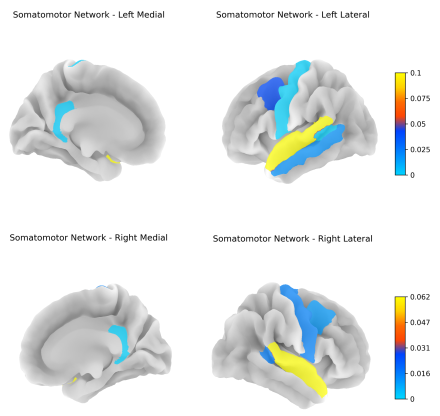

Fig. S6. Scales overlaid on parcellated surface mesh for Somato Motor Network

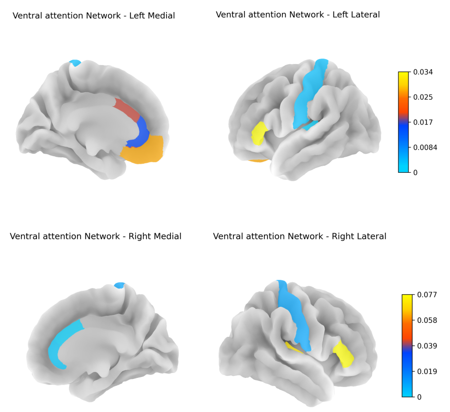

Fig. S7. Scales overlaid on parcellated surface mesh for Ventral Attention Network

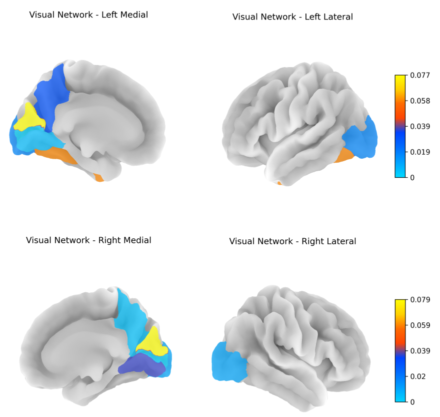

Fig. S8. Scales overlaid on parcellated surface mesh for Visual Network

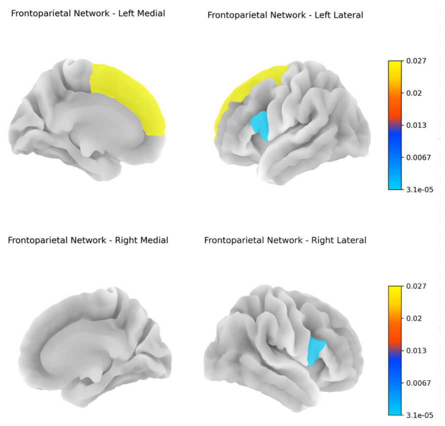

Fig. S9. Scales overlaid on parcellated surface mesh for Fronto Parietal Network

E. Perturbation Studies

Perturbation test helps us to know whether the model is learning the pattern and also its robustness to noise. We add Gaussian noise to the model input (SC) and observe how it affects the model performance. This is done in 2 ways:

1. Train the model on original data without noise and test the model on noisy input.

2. Train the model on noisy data and test on original data.

When we add noise to test SC, the structure is lost, and we expect the model performance to decrease marginally on test. When the model is trained on perturbed SC, we expect the model to learn the preserved pattern across subjects but also a performance decrease due to structure loss. The results are in the figures S10 and S11.

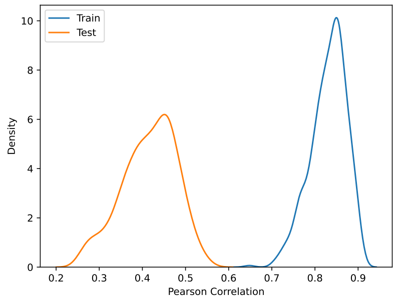

Fig. S10. The model trained on original data and tested on perturbed input. The Pearson's correlation drops by a large margin when input is perturbed.

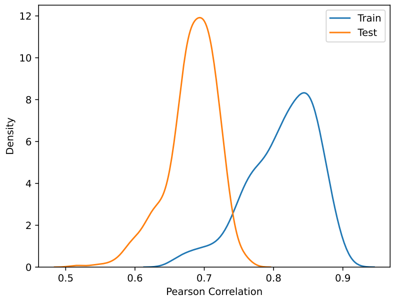

Fig. S11. Model trained on perturbed SC and tested on original SC. The Pearson's correlation drops for both train and test data.

F. Generalizability and Stability Experiments

To assess the generalizability of our model across different train-test splits, we conducted 5-fold cross validation (CV) experiments. The dataset was divided into 5 folds, with each fold containing around 211 subjects. For each run, the model was trained on 4 of these folds, leaving the remaining fold for testing. This cross-validation process was repeated until each fold had been used as the test set once. The performance results on cross-validation experiments are illustrated in Figure S12. Notably, the Pearson's correlation coefficient is close to 85%, indicating a strong positive relationship between the predicted and empirical FC across all folds, further validating the robustness and generalizability of the model.

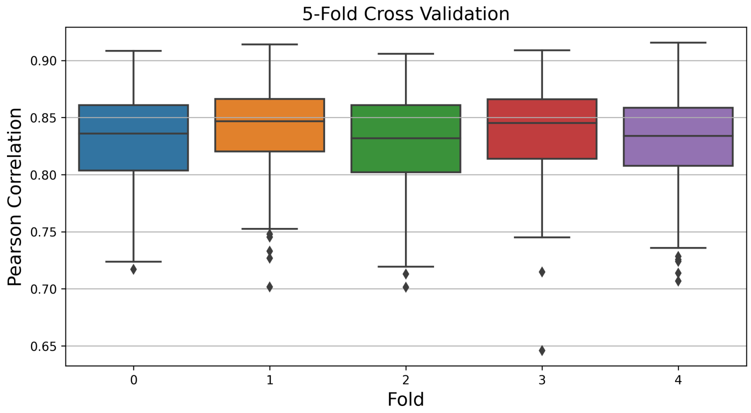

Fig. S12. 5-fold cross validation results. Each box-plot depicts results on the 210 subjects in the test-fold.

G. Derivation of Graph Laplacian

The Laplacian operator is defined as:

$$\Delta = \frac{\partial^2}{\partial x^2}$$

acting on functions $f(x)$, with analogous forms in other dimensions.

Let $f(x)$ be a function and suppose we aim to approximate the second derivative $\frac{d^2 f}{dx^2}(x)$. We can use a centered difference approximation as follows:

$$\begin{aligned} \frac{d^2 f}{dx^2} &\approx \frac{f'(x+\frac{\Delta x}{2}) - f'(x-\frac{\Delta x}{2})}{\Delta x} \\ &= \frac{1}{\Delta x}\left[\frac{f(x+\Delta x)-f(x)}{\Delta x} - \frac{f(x)-f(x-\Delta x)}{\Delta x}\right] \\ &= \frac{1}{(\Delta x)^2}\left[f(x+\Delta x) - f(x) + f(x-\Delta x) - f(x)\right] \\ &= \frac{1}{(\Delta x)^2}\left[f(x+\Delta x) + f(x-\Delta x) - 2f(x)\right]. \end{aligned}$$

Note that if we take $\Delta x = 1$, this approximation depends only on the function values at the integer points.

Now, consider the graph consisting of vertices on the integers of the real line, with edges connecting consecutive integers. For any function f defined on the vertices, the Laplacian can be computed as:

$$\Delta f(v_i) = f(v_{i+1}) + f(v_{i-1}) - 2f(v_i)$$

for any vertex v_i. The Laplacian is represented as an infinite matrix of the form:

$$\Delta f = \begin{bmatrix} \ddots & \ddots & \ddots & & \\ & 1 & -2 & 1 & 0 \\ & 0 & 1 & -2 & 1 \\ & & \ddots & \ddots & \ddots \end{bmatrix} \begin{bmatrix} \vdots \\ f(v_{i-1}) \\ f(v_i) \\ f(v_{i+1}) \\ \vdots \end{bmatrix}.$$

It is also important to note that this matrix is equivalent to the adjacency matrix minus twice the identity matrix. The number 2 represents the degree of each vertex, so we can express the Laplacian matrix as:

$$L = A - D,$$

where A is the adjacency matrix, and D is the diagonal matrix containing the degrees (also known as the degree matrix).

REFERENCES

1. C. Donnat, M. Zitnik, D. Hallac, and J. Leskovec, "Learning structural node embeddings via diffusion wavelets," in *Proceedings of the 24th ACM SIGKDD international conference on knowledge discovery & data mining*, (2018), pp. 1320–1329.
2. S. R. Oota, A. Yadav, A. Dash, *et al.*, "Attention-based fusion of multiple graphheat networks for structural to functional brain mapping," Sci. Reports **14**, 1184 (2024).
